# Investigating the Implementation of SMS and Mobile Messaging in Population Screening (the SIPS Study): Protocol for a Delphi Study

**DOI:** 10.2196/32660

**Published:** 2021-12-22

**Authors:** Amish Acharya, Gaby Judah, Hutan Ashrafian, Viknesh Sounderajah, Nick Johnstone-Waddell, Anne Stevenson, Ara Darzi

**Affiliations:** 1 Institute of Global Health Innovation Imperial College London London United Kingdom; 2 Public Health England London United Kingdom

**Keywords:** mobile messaging, digital communication, population screening, SMS, implementation

## Abstract

**Background:**

The use of mobile messaging, including SMS, and web-based messaging in health care has grown significantly. Using messaging to facilitate patient communication has been advocated in several circumstances, including population screening. These programs, however, pose unique challenges to mobile communication, as messaging is often sent from a central hub to a diverse population with differing needs. Despite this, there is a paucity of robust frameworks to guide implementation.

**Objective:**

The aim of this protocol is to describe the methods that will be used to develop a guide for the principles of use of mobile messaging for population screening programs in England.

**Methods:**

This modified Delphi study will be conducted in two parts: evidence synthesis and consensus generation. The former will include a review of literature published from January 1, 2000, to October 1, 2021. This will elicit key themes to inform an online scoping questionnaire posed to a group of experts from academia, clinical medicine, industry, and public health. Thematic analysis of free-text responses by two independent authors will elicit items to be used during consensus generation. Patient and Public Involvement and Engagement groups will be convened to ensure that a comprehensive item list is generated that represents the public’s perspective. Each item will then be anonymously voted on by experts as to its importance and feasibility of implementation in screening during three rounds of a Delphi process. Consensus will be defined a priori at 70%, with items considered important and feasible being eligible for inclusion in the final recommendation. A list of desirable items (ie, important but not currently feasible) will be developed to guide future work.

**Results:**

The Institutional Review Board at Imperial College London has granted ethical approval for this study (reference 20IC6088). Results are expected to involve a list of recommendations to screening services, with findings being made available to screening services through Public Health England. This study will, thus, provide a formal guideline for the use of mobile messaging in screening services and will provide future directions in this field.

**Conclusions:**

The use of mobile messaging has grown significantly across health care services, especially given the COVID-19 pandemic, but its implementation in screening programs remains challenging. This modified Delphi approach with leading experts will provide invaluable insights into facilitating the incorporation of messaging into these programs and will create awareness of future developments in this area.

**International Registered Report Identifier (IRRID):**

PRR1-10.2196/32660

## Introduction

In England, there are currently 11 different population screening programs that are involved in the detection of conditions as varied as abdominal aortic aneurysms and thalassemia [1]. Each year, over 10,000 lives are saved as a result of these different screening programs [2]. However, screening services are facing increasing challenges in the way they operate. Governance issues, poor interoperability of systems, and capacity concerns have negatively impacted on the effectiveness of services [3]. In 2018, these factors all contributed toward a national incident, where 120,000 women failed to receive their final mammogram appointment [4]. Moreover, there are now increasing concerns regarding falling uptake of screening invitations for conditions such as breast cancer across the United Kingdom [5]. Similar trends are also seen across the world, including in the United States and Canada [6].

Collectively, screening programs in England invite 15 million people annually; however, on average, only 10 million people take up the invitation [7]. This performance differs significantly between the individual programs. While diabetic eye screening is consistently well attended, uptake of cervical cancer screening is falling, with a 6.8% lower uptake in 2020 than in 2019 [8,9]. These trends are likely to be exacerbated as a result of COVID-19, in which screening services were ceased, leading to a backlog of millions of tests [10]. One way in which some services have attempted to address these concerns is by using mobile messaging.

Mobile messaging encompasses a range of text and multimedia messaging platforms through mobile devices and includes SMS and multimedia messaging service. It has been widely used in several industries, including banking and retail, and is often seen by the public as a less intrusive and more convenient means for communication, as compared to phone calls [11]. In one survey, 69% of respondents across all age groups wanted to be able to contact businesses by text [12]. Within health care, its use as an adjunctive means of communication between health care professionals and patients is also growing. Both primary and secondary care services use messaging in a variety of ways, including confirming appointments, as reminders, and for health promotion campaigns. Moreover, as with the COVID-19 pandemic, mobile messaging has been shown to be an effective and acceptable means of providing public health information, when direct access to hospital or health care services is limited [13]. Within screening programs, the use of mobile messaging, predominantly SMS, is well established as a reminder tool. SMS reminders in breast cancer screening have been shown to increase attendance by 5% [14]. Furthermore, research has shown that by altering the content of these messages it may be possible to improve declining uptake rates [15]. The formal cross-program adoption of mobile reminders is, therefore, one of the foremost recommendations in the United Kingdom’s Independent Review of Adult Screening Programmes [7].

Adopting mobile messaging, whether for reminders or health promotion in this context, however, provides a unique set of challenges. Unlike individual general practitioner (GP) practices, regional screening hubs will send hundreds of thousands of messages annually [16]. These large target populations consist of a diverse range of people with differing health literacy levels, awareness of screening services, and communication needs. There are already concerns that mobile messaging may exacerbate inequalities among different socioeconomic groups, thereby creating a technological divide; on a population scale, this possibility is much greater [17]. Furthermore, in some programs, screening is not undertaken through the GP practice or a known health care service, but by a screening hub. An individual may be contacted by a service they have never interacted with previously and, therefore, may lack trust in the provenance and content of the message. Moreover, if messages are not sent from the GP practice, this also introduces potential security issues that need consideration, as it is not always possible to verify mobile numbers of previous nonattenders or new invitees. Above all, screening is a choice, and a clear balance is needed between facilitating patients to attend screening, but not coercing them to attend. The message content, therefore, should also differ from messaging for outpatient appointments, which may have been requested directly by the patient or by proxy. There is, however, a paucity of guidance about how screening services can use mobile messaging appropriately.

Of the few published frameworks on the use of mobile messaging in health care, the predominant focus is on the use of mobile messaging in a single service (eg, a GP practice) or on the provision of general content-based recommendations only [18,19]. Therefore, they fail to provide guidance on the aforementioned breadth of areas of contention faced by population screening programs. The aim of this study is to use a modified Delphi approach to determine the opinions of experts in the fields of preventative care, screening, health communications, and academia, and to draw a consensus on the key issues and ways mobile messaging can be implemented into population screening. Through this process, we will aim to create an expert-derived list of current recommendations for services, as well as highlight areas to inform the future direction of mobile messaging in this context. This study is registered on the Open Source Framework as the SMS and Mobile Messaging in Population Screening (SIPS) study [20].

## Methods

### Study Design

The study was developed using a modified Delphi approach, which aims to integrate opinions from stakeholders and experts in the field [21]. Delphi exercises have been used in a variety of health care and technology contexts, such as medical artificial intelligence reporting guidelines [22]. Unlike other consensus group methods, such as the nominal group technique (NGT), Delphi methods lend themselves to being conducted remotely, which is a necessity given current social distancing constraints [23]. Moreover, unlike the NGT, the Delphi technique can be used to synthesize the viewpoints of a large group of experts from a variety of backgrounds, as is required with several different population screening programs [24]. While there are concerns regarding the reproducibility of the results from Delphi studies, the attrition during the process, and issues with validity, it remains one of the most widely used means of deriving expert-level consensus [25]. One of the reasons for these criticisms is the paucity of best practice guidance or standardization when undertaking Delphi methodology. It is accepted, however, that through an iterative process, in which individuals are sequentially asked to rate items and guided to amend these ratings in light of feedback from the collective response, the Delphi process can effectively derive consensus. By recruiting individuals with a breadth of related experience and ensuring the anonymity of responses, it enables a wide range of results applicable in several allied fields to be elicited, without a single voice or group dominating [26]. This is particularly important with population screening in which each program has its own messaging infrastructure, delivery system, and needs. If the results were to favor one of the groups more than the other, ultimately, it would make the findings of this study less applicable.

**Figure 1:**
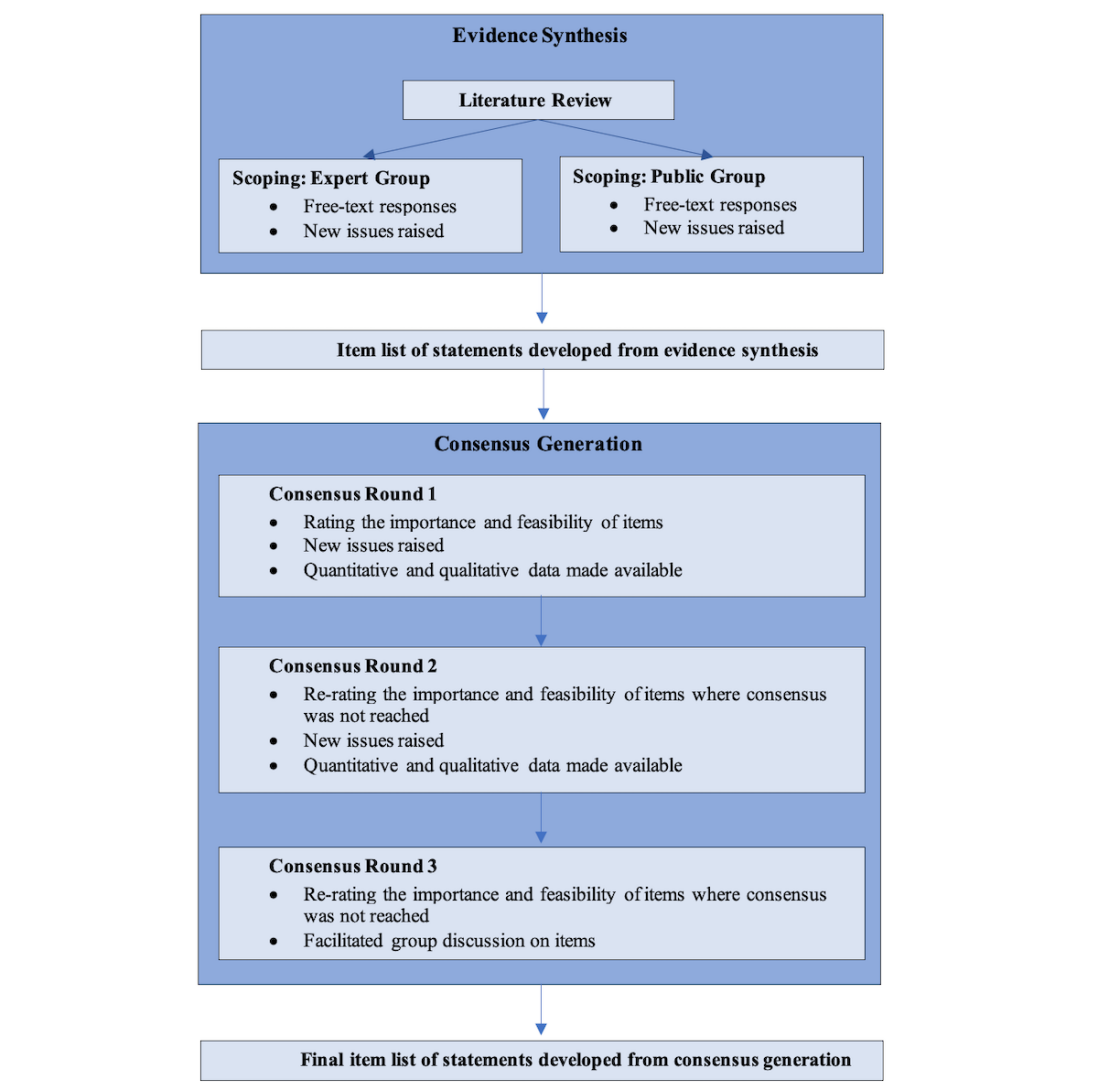
Diagram demonstrating the stages of the study, including the primary outcome.

The procedure for this modified Delphi study will be undertaken in two parts: evidence synthesis and consensus generation (Figure 1).

### Ethics

The Institutional Review Board at Imperial College London has granted ethical approval for this study (reference 20IC6088). All public and expert participants will be required to provide informed consent for each part of the study and have the freedom to withdraw consent at any stage.

### Part 1: Evidence Synthesis

#### Overview

To develop a list of items of interest for experts to deliberate on for the consensus rounds, a literature review will be undertaken. This will be supplemented by an online expert and public scoping exercise.

#### Literature Background

A review of literature published from January 1, 2000, to October 1, 2021, in both academic and nonacademic forums was undertaken to inform the development of this protocol and determine the areas that require addressing in the consensus. A search of PubMed, MEDLINE, Embase, and Google Scholar databases was undertaken using various terms, including the following: “mass screening,” “population screening,” “abdominal aortic aneurysm,” “diabetic retinopathy,” “cervical cancer,” “cervical smear,” “mammography,” “colonoscopy,” “sigmoidoscopy,” “newborn hearing,” “thalassemia,” “sickle cell,” “congenital abnormalities,” “chorionic villus sampling,” “amniocentesis,” “lung cancer,” “chest x ray” in conjunction with “mobile, cell, phone messaging,” “short message service,” “multimedia messaging service,” and “text messages.” Terms were combined using the Boolean operators “OR” and “AND,” as appropriate. A full list of search terms can be found in Multimedia Appendix 1. Duplicates were removed and studies were imported into the review software Covidence. Two authors (AA and VS) independently screened titles and abstracts to check for relevance to the research question. Disagreements were discussed until resolution was reached.

Additional articles relevant to health care messaging were also found using grey literature, including publications from health care organizations, such as Public Health England, the National Institute for Health and Care Excellence, and the US Food and Drug Administration. Again, these documents were screened by the two independent authors, with disagreements discussed. Included articles were reviewed and the data were extracted to provide evidence for higher-order domains of interest relating to messaging in screening, provide individual items for inclusion into the scoping stage, and highlight areas of contention.

#### Scoping Exercise: Experts

An online scoping questionnaire based on evidence generated from the literature review will be undertaken to determine whether there were any notable areas of omission and areas for further consideration, and to help generate an item list for the consensus round. This scoping questionnaire will be framed according to six domains of interest elicited from the literature review: content, timing, delivery, evaluation, security, and future considerations. The scoping exercise is designed to elicit unrestricted free-text responses from individuals, with prompts given under each of the six headings to guide answers. Experts will be given the freedom to respond in any way that suits them, including providing examples of different types of messages and links to evidence to support their point. The scoping questionnaire will be delivered online independently to a group of experts (see Expert Recruitment section below), who will be encouraged to use their experience and appreciation of available guidance.

Free-text responses will then be examined using a thematic approach. Authors will first familiarize themselves with responses, sorting them according to the higher domains to which they pertained. Both authors will then independently code each response and derive recurring subthemes and disagreements. These subthemes will be discussed among the wider group of authors, and an item list will be generated. Through discussion, disagreements will be resolved on whether an item should be included or regarding the wording of a particular item.

#### Patient and Public Involvement and Engagement Group

Population screening messages directly impact the public. It is, therefore, important to appreciate and include the experience of members of the public, in order to highlight areas that may not have been previously addressed by experts or the existing literature. Members of the public will be recruited through the existing affiliations of authors, as well as via the online health and social care research platform VOICE. Participants will only be included if they are over 18 years of age and based within England at the time of the study. Members of the public will be asked to provide their own experiences with health care messaging within screening programs through free-text responses. This will mirror the themes from the expert questionnaire and will include jargon-free prompts, developed in discussion with representatives of the VOICE platform. Input from this Patient and Public Involvement and Engagement (PPIE) group will be used to ensure that items are relevant to the public, with the consensus list amended following this review. The responses will be mapped to the finalized item list derived from the expert consensus by two independent authors. If an item does not map onto an existing item, it will be added as an additional item under the appropriate higher domain. Quantitative data (ie, frequencies [eg, 50% stated that item X was important] and rankings [eg, item X is more important than Y]) and qualitative data (ie, summarizing quotes) will also be elicited from the public free-text responses. These will be included under the corresponding item during the consensus generation process. These public responses will be used to provide further guidance for the experts during the consensus rounds and to ensure the public perspective is included.

### Part 2: Consensus Generation

#### Expert Recruitment

Experts recruited to participate in the scoping and consensus stages will be from a broad range of intersecting specialties, such as academia, clinical medicine, screening administration, health care consultancy, behavioral science, industry, and public and population health. Input from a diverse array of sectors ensures that findings are widely relevant and reduces the chance of omission of important areas of discussion. To be included, experts require professional experience in one of the 11 active screening programs in the United Kingdom, or in the Targeted Lung Health Checks program, which is currently being piloted. While no strict definition of an “expert” is used, authors will recruit only individuals who hold prominent regional or national roles within their field, have evidence of impact within that area, or have conducted prominent academic research into screening programs. As this study focuses on UK screening programs, and because other countries conduct screening differently, an international group of experts will not be contacted at this stage. In keeping with previous Delphi studies, no sample size calculation has been conducted, as the resultant consensus findings are related to the experience of the individuals, as opposed to the number recruited [27,28]. We plan to recruit a minimum of 20 experts across the aforementioned specialties, as this number has previously been shown to provide reliable judgments [23]. Invited individuals will be agreed on through discussion between all authors, with disagreements discussed until these have been resolved.

#### Consensus Rounds

Two rounds will be conducted in order to gain consensus on the items. Specifically, participants will be asked to rate (1) the importance and (2) the feasibility of incorporating each item into mobile messaging for population screening. Importance is defined as a characteristic that is fundamental to the effective use, governance, or development of messages from or for the screening service. Feasibility is defined as a message characteristic that can be easily incorporated into the current system without significant cost, time, or logistical difficulty. Each item will be listed under one of the six higher domains. For each item, experts will respond on a 5-point Likert scale for importance (ie, 1 = extremely important, 2 = important, 3 = neither important nor unimportant, 4 = unimportant, and 5 = extremely unimportant) and feasibility (eg, 1 = absolutely feasible, 2 = feasible, 3 = neither feasible nor unfeasible, 4 = unfeasible, and 5 = absolutely unfeasible). Additionally, as not all items will be relevant to each screening program, a sixth option of “not relevant” will be added in. Question logic will ensure that if an individual selects this option, they will not be able to rate the feasibility or importance of that particular item. Within the first consensus round, data from the previous PPIE group and the National Cyber Security Centre will be provided to experts to aid their decision making.

While there is no formal accepted definition of consensus, within this study, we have predefined the threshold at 70% [29]. Items that more than 70% of experts deem “extremely unimportant” or “unimportant” and “extremely unfeasible” or “unfeasible” (scores of 4 or 5) will be excluded from subsequent rounds. Items that achieve more than 70% consensus as “extremely important” or “important” and “extremely feasible” or “feasible” (scores of 1 or 2) will be included in the final core item list for discussion. Items that do not reach these thresholds of consensus will be put forward to the next consensus round. Furthermore, participants will have the option to add free-text responses or suggestions for further items to be included, which will be available to vote on in the next consensus round. This second consensus round will have the same format as the first, but will include new items suggested from round 1. Moreover, the aggregated responses from round 1 will be made available for each item. Through this feedback on the collective response, it is hoped that consensus on the remaining items can be achieved. Items will be treated similarly to the first round, with those deemed important and feasible put forward to the final core item list. Items that are found to be important but not feasible, following the second consensus round, will be considered “desirable” and will be placed on a separate list for discussion at the consensus meeting. However, items that are voted to be feasible but not important will be excluded, given their lack of utility for screening services.

#### Consensus Meeting

The final round will be a consensus meeting, held online due to the current social distancing constraints. This meeting will be facilitated by the authors of the study in order to provide a structured means of interaction between individuals and to enable all members to have their voices heard. Furthermore, the use of a consensus meeting provides experts the opportunity to provide justification of their viewpoint and seek clarification where necessary. Previous research has highlighted this modification of the original Delphi methodology as more effective and collaborative [30,31]. The aim of this meeting is to develop a finalized list of important and feasible items to guide mobile messaging use in population screening programs. To achieve this objective, attendees at the meeting will first be presented with the results from the second consensus round. Any item that has not reached a consensus will then be discussed and voted on again using the 5-point Likert scales for importance and feasibility. All items that have reached a positive consensus at any point throughout the process will then be discussed further for consideration into the final recommendation. This recommendation will consist of items that experts agree are important and currently feasible for incorporating into screening program messaging. A separate list of items that are considered important but not feasible will also be included. These will be considered aspirational or desirable qualities that could guide future developments in this area.

### Data Availability

Data sharing is not applicable to this paper as no data sets were generated or analyzed during this study.

## Results

The literature review is underway. A final list of feasible and important items to consider in population screening programs will be published in 2022. In addition to publishing this work in a peer-reviewed journal, we aim to make this work available to screening services in England through presentations and online posts. In this way, we hope stakeholders will easily have access to, and be able to benefit from, the lessons derived from this study.

## Discussion

The use of mobile messaging has grown significantly across health care services, especially given the COVID-19 pandemic. While its use in population screening programs (eg, as reminders) has been supported by policy makers and patients alike, the implementation of messaging remains challenging. Screening programs pose a unique set of challenges to the use of messaging regarding the content, timing, delivery, evaluation, and security of messages. Moreover, as the digital literacy of the population continues to grow, how future technologies can be integrated must also be considered. This is particularly important as messaging functionality develops further, with the increasing penetrance of technologies such as rich communication services. These services allow for more complex messaging (eg, sending location services and higher-resolution multimedia) to be undertaken and, therefore, it is increasingly important for screening services to have guidance on how best to implement them [32]. There is, however, a paucity of information regarding the application of messaging systems in screening. We hope this modified Delphi approach with leading experts will provide invaluable initial insights into facilitating the incorporation of messaging into these programs and creating awareness of future developments in this area. Furthermore, we hope our findings will enable comparisons with screening services internationally, in order to determine the most effective means of using mobile messaging to facilitate attendance.

## Appendix

Multimedia Appendix 1 Supplementary material, including search strategy for evidence generation.

## Abbreviations

GP: general practitioner
NGT: nominal group technique
NIHR: National Institute for Health Research
PPIE: Patient and Public Involvement and Engagement
SIPS: SMS and Mobile Messaging in Population Screening

## References

[1] (2021). NHS population screening explained. Public Health England.

[2] (2019). Modern screening can be more personalised and convenient to save lives says new report. NHS England.

[3] (2019). Adult health screening. UK Parliament.

[4] (2018). Internal PHE investigation into the national breast screening incident of 2018. Public Health England.

[5] (2021). Breast Screening Programme, England 2019-20. NHS Digital.

[6] Crawford J, Ahmad F, Beaton D, Bierman AS (2016). Cancer screening behaviours among South Asian immigrants in the UK, US and Canada: A scoping study. Health Soc Care Community.

[7] Richards M (2019). Report of the Independent Review of Adult Screening Programmes in England.

[8] Public Health England (2020). Diabetic eye screening: 2018 to 2019 data. GOV.UK.

[9] (2021). Cervical Screening Programme - Coverage statistics. NHS Digital.

[10] Ho KMA, Banerjee A, Lawler M, Rutter MD, Lovat LB (2021). Predicting endoscopic activity recovery in England after COVID-19: A national analysis. Lancet Gastroenterol Hepatol.

[11] Martinengo L, Spinazze P, Car J (2020). Mobile messaging with patients. BMJ.

[12] Goodwin H (2019). Texting report reveals 69 percent of consumers want to contact businesses via text. Business Wire.

[13] Lee M, You M (2021). Effects of COVID-19 emergency alert text messages on practicing preventive behaviors: Cross-sectional web-based survey in South Korea. J Med Internet Res.

[14] Kerrison RS, Shukla H, Cunningham D, Oyebode O, Friedman E (2015). Text-message reminders increase uptake of routine breast screening appointments: A randomised controlled trial in a hard-to-reach population. Br J Cancer.

[15] Huf S, Kerrison RS, King D, Chadborn T, Richmond A, Cunningham D, Friedman E, Shukla H, Tseng F, Judah G, Darzi A, Vlaev I (2020). Behavioral economics informed message content in text message reminders to improve cervical screening participation: Two pragmatic randomized controlled trials. Prev Med.

[16] (2020). Breast Screening Programme, England 2018-19. NHS Digital.

[17] Marler W (2018). Mobile phones and inequality: Findings, trends, and future directions. New Media Soc.

[18] (2020). Screening text message principles. Public Health England.

[19] (2021). Text message communication in general practice. The Medical Defence Union (MDU).

[20] Acharya A, Judah G, Ashrafian H, Sounderajah V, Johnstone-Waddell N, Stevenson A, Darzi A (2021). The SMS and mobile messaging In Population Screening (SIPS) study. Open Science Framework.

[21] McKenna HP (1994). The Delphi technique: A worthwhile research approach for nursing?. J Adv Nurs.

[22] Sounderajah V, Ashrafian H, Aggarwal R, De Fauw J, Denniston AK, Greaves F, Karthikesalingam A, King D, Liu X, Markar SR, McInnes MDF, Panch T, Pearson-Stuttard J, Ting DSW, Golub RM, Moher D, Bossuyt PM, Darzi A (2020). Developing specific reporting guidelines for diagnostic accuracy studies assessing AI interventions: The STARD-AI Steering Group. Nat Med.

[23] Humphrey-Murto S, Varpio L, Wood TJ, Gonsalves C, Ufholz L, Mascioli K, Wang C, Foth T (2017). The use of the Delphi and other consensus group methods in medical education research. Acad Med.

[24] McMillan SS, King M, Tully MP (2016). How to use the nominal group and Delphi techniques. Int J Clin Pharm.

[25] Lam K, Iqbal FM, Purkayastha S, Kinross JM (2021). Investigating the ethical and data governance issues of artificial intelligence in surgery: Protocol for a Delphi study. JMIR Res Protoc.

[26] Boulkedid R, Abdoul H, Loustau M, Sibony O, Alberti C (2011). Using and reporting the Delphi method for selecting healthcare quality indicators: A systematic review. PLoS One.

[27] Wong HS, Curry NS, Davenport RA, Yu L, Stanworth SJ (2020). A Delphi study to establish consensus on a definition of major bleeding in adult trauma. Transfusion.

[28] Macfarlane L, Owens G, Cruz BDP (2016). Identifying the features of an exercise addiction: A Delphi study. J Behav Addict.

[29] Diamond IR, Grant RC, Feldman BM, Pencharz PB, Ling SC, Moore AM, Wales PW (2014). Defining consensus: A systematic review recommends methodologic criteria for reporting of Delphi studies. J Clin Epidemiol.

[30] Eubank BH, Mohtadi NG, Lafave MR, Wiley JP, Bois AJ, Boorman RS, Sheps DM (2016). Using the modified Delphi method to establish clinical consensus for the diagnosis and treatment of patients with rotator cuff pathology. BMC Med Res Methodol.

[31] Gustafson DH, Shukla RK, Delbecq A, Walster G (1973). A comparative study of differences in subjective likelihood estimates made by individuals, interacting groups, Delphi groups, and nominal groups. Organ Behav Hum Perform.

[32] (2021). Tapping into the benefits of rich communication services. Healthcare Communications.

