# Supplementary material for: Investigating the Implementation of SMS and Mobile Messaging in Population Screening (the SIPS Study): Protocol for a Delphi Study
Source: JMIR Res Protoc. 2021 Dec 22;10(12):e32660. doi: 10.2196/32660 (PMC8734915; doi:10.2196/32660)
Supplement: Multimedia Appendix 1 [file resprot_v10i12e32660_app1.docx]

*Multimedia Appendix 1. Supplementary Material*

*Exemplar Search Items for Database*

| *Medline* | | |
| --- | --- | --- |
| 1 | (antenatal screening[MeSH Terms]) OR (cancer screening[MeSH Terms])) OR (cancer screening test[MeSH Terms])) OR (mass screening[MeSH Terms]) OR (screen[MeSH Terms]) | 230,441 |
| 2 | Population screening programme OR population screen* | 487,186 |
| 3 | 1 or 2 | 280,513 |
| 3 | (papanicolaou test[MeSH Terms]) OR (papanicolaou smear[MeSH Terms]) OR (cervical smear[MeSH Terms]) OR (vaginal smear[MeSH Terms]) OR (pap* smear) OR (smear test*)) OR (cervical cancer screen*) OR (cervical screen*) (pap* test)) OR (smear test*) | 77,482 |
| 4 | (mammographies[MeSH Terms]) OR (mammography[MeSH Terms]) OR (breast cancer screening) OR (x ray screening)) OR (mamm*) OR (breast screen*) | 1,583,976 |
| 5 | (procedures, sigmoidoscopic surgical[MeSH Terms]) OR (bowel cancer screen*)) OR (bowel screen*) OR (colorectal cancer screen*) OR (colorectal screen*) OR (f?ecal occult blood test) OR (f?ecal immunochemical test) OR OR (f?ecal immuno-chemical test) OR OR (sigmoidoscop*) OR (colonoscop*) OR (flexisigmoidoscopy)) OR (bowel scope)) OR (colo-rectal cancer screen*)) OR (colo-recal screen*) | 234,669 |
| 6 | (neonatal screening[MeSH Terms]) OR (prenatal screening[MeSH Terms])) OR ((neonat* or newborn or pregnan* or prenatal or antenatal or pr nata on ante natal or fetal or fetal or fetus or foetus) OR (newborn and infant physical exam* OR NIE OR (newborn blood spot) OR neonatal blood spot) OR (f?etal abnormality test*)) OR (f?etal abnormality screening) OR (ultrasound screen*)) OR (ultrasound assess*)) OR (structural survey)) OR (chorionic vill* sampl*)) OR (amnio*) OR (nuchal translucency) OR (ultra-son*) | 3,411,9110 |
| 7 | ("diabetic retinopathy/diagnosis"[MeSH Terms]) OR (diabet* eye screen*) (diabet* eye test*) OR (diabet* maculo*) OR (diabet* vision screening) | 19,727 |
| 8 | ((pregnan* or antenatal on ante natal) and (div of human immunodeficiency virus or hepatitis or hepb or hep b or syphilis or sexually transmitted infection* of sexual transmitted disease*) and screening). | 6,363 |
| 9 | (abdominal aortic aneurysm[MeSH Terms]) OR (AAA)) OR (aneurysm screen*)) OR (aortic screen*) OR (aortic aneurysm screening) | 182,224 |
| 10 | 1-9 | 4,278021 |
|  | (messaging, text[MeSH Terms]) OR (mobile messag*) OR (SMS) OR (healthcare messag*) OR (patient messag*)) OR (mobile communication) OR (cell messag*)OR (cell communication) OR (WhatsApp) OR (MMS) OR (multimedia messaging) OR (bidirectional messaging) OR (bi-directional messaging) OR (App* messag*) | 144,610 |
| 11 | 10+11 | 16,170 |
| 12 | Limit Date  Limit Language | 8,549 |
